# Evaluation of a DNA Aβ42 vaccine in adult rhesus monkeys (*Macaca mulatta*): antibody kinetics and immune profile after intradermal immunization with full-length DNA Aβ42 trimer

**DOI:** 10.1186/s13195-017-0257-7

**Published:** 2017-04-26

**Authors:** Doris Lambracht-Washington, Min Fu, Pat Frost, Roger N. Rosenberg

**Affiliations:** 1 Department of Neurology and Neurotherapeutics, UT Southwestern Medical Center, 5323 Harry Hines Boulevard, Dallas, TX 75390-8813 USA; 2 Texas Biomedical Research Institute, San Antonio, TX USA; 3 Alzheimer’s Disease Center, UT Southwestern Medical Center, Dallas, TX 75390 USA

**Keywords:** Alzheimer’s disease, Immunotherapy, Nonhuman primates, DNA vaccination, Amyloid-β, Antibody response, Th2 immune response

## Abstract

**Background:**

Aggregated amyloid-β peptide 1–42 (Aβ42), derived from the cellular amyloid precursor protein, is one of the pathological hallmarks of Alzheimer’s disease (AD). Although active immunization against Aβ42 peptide was successful in AD mouse models and led to removal of plaques and improved memory, a similar clinical trial in humans (Aβ42 peptide immunization with QS-21 adjuvant) was stopped in phase II, when 6% of the treated patients developed encephalitis. Currently ongoing passive immunizations with the injection of preformed monoclonal antibodies against different epitopes within the Aβ_1–42_ peptide, which do not lead to activation of the immune system, have shown some effects in slowing AD pathology. Active DNA Aβ42 immunizations administered with the gene gun into the skin are noninflammatory because they activate a different T-cell population (Th2) with different cytokine responses eliciting a different humoral immune response. We present our findings in rhesus macaques that underwent the DNA Aβ42 immunization via gene gun delivery into the skin.

**Methods:**

Six rhesus monkeys received two different doses of a DNA Aβ42 trimer vaccine. The humoral immune response was analyzed from blood throughout the study, and cellular immune responses were determined in peripheral blood mononuclear cells (PBMCs) after three and six immunizations.

**Results:**

DNA Aβ42 trimer immunization led to high titer antibody responses in the nonhuman primate (NHP) model. Antibodies generated in the rhesus monkeys following DNA Aβ42 immunization detected amyloid plaques consisting of human Aβ42 peptide in the brain of the triple-transgenic AD mouse model. T-cell responses showed no interferon (IFN)-γ- and interleukin (IL)-17-producing cells from PBMCs in Enzyme-Linked ImmunoSpot assays after three immunization time points. At six immunization time points, IFN-γ- and IL-17-producing cells were found in immunized animals as well as in control animals and were thus considered nonspecific and not due to the immunization regimen. IFN-γ and IL-17 secretion in response to Aβ42 peptide restimulation became undetectable after a 3-month rest period.

**Conclusions:**

Intradermal DNA Aβ42 immunization delivered with the gene gun produces a high antibody response in NHPs and is highly likely to be effective and safe in a clinical AD prevention trial in patients.

## Background

Alzheimer’s disease (AD) is characterized by two pathological hallmarks: (1) senile plaques consisting of aggregated amyloid-β peptide 1–42 (Aβ42) and (2) neurofibrillary tangles consisting of aggregated tau proteins. Immunotherapy has the rationale that immunization induces an immune response that is able to clear plaque and tangles, which has high potential to treat these two hallmarks and has been successful in mouse AD models [1–3].

In a first clinical trial in which full-length Aβ_1–42_ peptide was stopped in 2002 in phase II, when 6% of the immunized patients developed meningoencephalitis that was due to inflammatory T-cell responses likely caused by the adjuvant, which was used to enhance the immune response and antibody production [4–7]. The clinical evaluations of the patients performed later showed that immunizations with Aβ42 peptide initiated the production of anti-Aβ42 antibodies and led to reduction of Aβ levels in brain and lesser plaque counts in the immunized patients. Furthermore, the level of amyloid removal showed a clear relationship to the titers of anti-Aβ42 antibodies present in the patients analyzed [8, 9]. Thus, Aβ42 immunotherapy and anti-Aβ antibodies have high potential to be effective in the removal of excess amyloid from the brain in patients with AD.

To avoid the inflammatory cellular side effects, large numbers of ongoing clinical trials are using passive immunizations with preformed anti-Aβ monoclonal antibodies [10–16]. One of the most recent clinical trials (of aducanumab in a double-blind, placebo-controlled phase Ib randomized trial [16]) in patients with prodromal or mild AD using a fully human monoclonal antibody selectively targeting aggregated Aβ, including soluble oligomers and insoluble fibrils, showed that following 1 year of monthly intravenous infusions of this antibody, brain Aβ had been reduced in a dose- and time-dependent manner. Furthermore, the investigators were able to show a slowing of clinical decline as measured by Clinical Dementia Rating and Mini Mental State Examination scores. [16]. However, passive immunizations carry different side effects, such as brain microhemorrhage [17], which was also found in this recent trial. Passive immunization is expensive, which makes the distribution and availability to large patient populations difficult. Active DNA immunization is in general less expensive, and it has been shown by us and others that DNA immunization into the skin results in sufficient antibody responses and a noninflammatory cellular immune response [18–28]. With recent progress in this area of immunization in the clinic, DNA immunizations are no longer restricted to preclinical studies in rodents and veterinary practice [29–33].

The genome sequence of the rhesus macaque shares about 93% of its sequence with the human genome and is thus an evolutionarily close relative to humans. Successful DNA Aβ42 immunization in a nonhuman primate (NHP) model, the rhesus monkey (*Macaca mulatta*), will thus provide valuable information about the immune responses and possible side effects if this type of immunotherapy is given to human patients. We have previously shown that DNA Aβ42 immunization was effective in eliciting an antibody response in aged mice and rabbits [24, 34]. Our working hypothesis is that when DNA Aβ42 immunization results in good antibody responses in the rhesus monkey, it is highly likely that this vaccine will result in a similar humoral immune response in humans as well. DNA Aβ42 immunization in rhesus monkeys is an important step prior to proceeding to a clinical trial. Therefore, we tested our DNA Aβ42 vaccination protocol in a group of adult rhesus macaques to show its effects on the immune system, including the anticipated antibody responses and unwanted cellular side effects. Positive outcome measures derived from these experiments indicative of effectiveness and safety are high antibody titers (1:25,000) without accompanying inflammatory interferon (IFN)-γ- and interleukin (IL)-17-producing T-cell responses.

## Methods

### Animals and immunizations

**Fig. 1:**
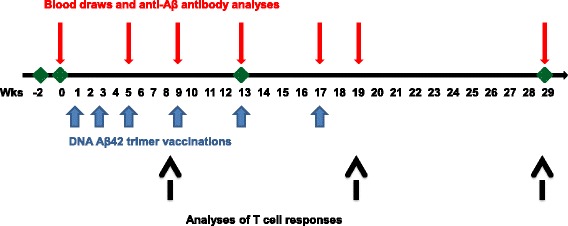
Illustration of the time line of the performed experiments and analyses of the immune responses in rhesus macaques. *A*β*42* Amyloid-β peptide 1–42

Eight 6- to 10-year-old rhesus macaques (four females, four males, weighing 4.5–11 kg) had been selected for this study (Southwest National Primate Research Center, San Antonio, TX, USA). The macaques had not been used in any research studies prior to this study. During the period of the study, the animals were housed in groups of two and were monitored daily for signs of illness or distress. The macaques were randomly separated in two groups (three animals/group). One group received a high-dose immunization regimen of 16 μg of DNA per immunization time point, and the other group received a low-dose regimen of 8 μg of DNA per immunization time point. Two animals served as controls to evaluate responses in nonimmunized macaques. The intradermal DNA immunizations with plasmid DNA encoding Aβ42 trimer were performed into the skin of the upper inner arm using the Helios gene gun (Bio-Rad Laboratories, Hercules, CA, USA). In brief, DNA-coated gold particles were injected into the skin with a helium pressure of 400 psi for a total of six immunization time points. The first three immunizations were done in biweekly intervals, followed by three vaccinations in monthly intervals. Three months following these six immunizations, a final blood draw was performed to analyze antibody half-life. The experimental schedule is based on a possible schedule for treatment of patients with early AD in a future clinical trial and is illustrated in Fig. 1. The three initial immunizations in biweekly intervals will initiate and boost an immune response; the following three immunizations in monthly intervals will maintain and strengthen the humoral antibody response. Animal use for this study was approved by the Institutional Animal Care and Use Committee of the Texas Biomedical Research Institute.

### Antibodies and peptides

The anti-Aβ42 immune response was measured with a panel of antimonkey immunoglobulin G (IgG), IgM, and IgA antibodies (Rockland Immunochemicals, Limerick, PA, USA) and antihuman IgG, IgG1, IgG2, and IgG4 antibodies (BD Biosciences, San Jose, CA, USA). An unlabeled rhesus monkey IgG antibody (SouthernBiotech, Birmingham, AL, USA) was used as a standard antibody to determine the anti-Aβ IgG immune response. Aβ peptides and other peptides used in this study had been purchased from rPeptide (Bogart, GA, USA), AnaSpec (Fremont, CA, USA), New England Peptide (Gardner, MA, USA), Bachem (Bubendorf, Switzerland), and American Peptide Company (Sunnyvale, CA, USA).

### Plasma collections

Blood was collected prior to the first immunization; after the second, third, fourth, and fifth immunizations; and 2 and 8 weeks past the sixth immunization. Antibody levels were determined from all blood samples. Blood chemistry and complete blood count (CBC) were determined from samples prior to the first immunization, after the fourth immunization, and from blood samples drawn 8 weeks past the fifth immunization. Lymphocytes from blood were isolated by density separation centrifugation using Lympholyte® Mammal Cell Separation Media (Cedarlane, Burlington, ON, Canada). Tissue culture was performed as previously described [22–24].

### Antibody enzyme-linked immunosorbent assay

Aggregated Aβ_1–42_ peptide was prepared as described previously [19]. Briefly, the peptide was prepared by adding 250 μl of PBS, pH 7.4, to 1 mg of lyophilized Aβ_1–42_ (counterion trifluoroacetic acid), followed by an overnight incubation at 37 °C. Anti-Aβ antibodies in rhesus plasma were measured according to standard procedures. High-binding 96-well plates were coated with human Aβ_1–42_ peptide (2 μg/ml) in 50 mM carbonate buffer, pH 9.6, overnight at 4 °C. Standard curves were included by binding of serial dilutions of an unlabeled rhesus monkey IgG antibody to the enzyme-linked immunosorbent assay (ELISA) plates. Plasma samples were diluted 1:400 and analyzed in triplicates. ELISAs were repeated three or four times, and data from one representative ELISA for the different time points are shown.

ELISAs for antibody titers in rhesus monkey plasma were performed according to standard procedures. The titers of antibodies were calculated as the reciprocal of the highest serum dilution that gave a reading twice the baseline of a 450-nm optical density (OD_450_) of 0.2. Plasma samples were serially diluted up to 1:50,000 from an initial dilution of 1:100. Secondary isotype antibodies used had been cross-adsorbed with rhesus IgG, IgA, or IgM, respectively (Rockland Immunochemicals).

For the antibody epitope studies, all Aβ peptides (1–42, 1–16, 6–20, 17–31, 22–35, 23–42) were used in 1 μM dilutions to compensate for the different lengths of the amino acid sequences and the number of epitopes available on the ELISA plate for the antibody binding. Epitope binding of IgG, IgA, and IgM antibody isotypes was analyzed.

For the antibody specificity studies, peptides (Aβ_1–42_, scrambled Aβ42, islet amyloid peptide 22–27, tau_275–305_, ovalbumin [OVA_265–280_], prion protein [Prp_106–140_], serum amyloid P component [SAP], and tetanus toxin [TTX_830–844_]) were used in dilutions of 2 μg/ml. Binding of IgG and IgA antibodies was analyzed from plasma samples diluted 1:500.

### Cytokine Enzyme-Linked ImmunoSpot and ELISA assays

Enzyme-Linked ImmunoSpot (ELISPOT) assays to determine frequencies of cytokine-secreting cells were performed according to standard procedures and as previously described using commercial available antibody sets for rhesus and human IFN-γ, IL-17, and IL-4 (Mabtech, Stockholm, Sweden) [21–23]. For maximal T-cell stimulation, concanavalin A (ConA; 2.5 μg/ml) or an antirhesus CD3 antibody (Mabtech) were used in 48-h cultures. IFN-γ concentrations from cell culture supernatants were measured using a commercially available rhesus monkey IFN-γ ELISA kit (Mabtech).

### Analysis of cell proliferation by carboxyfluorescein succinimidyl ester dilution

Cells for labeling were resuspended in PBS and immediately mixed with an equal volume of carboxyfluorescein succinimidyl ester (CFSE) diluted in PBS (1:1). The cells were incubated for 5 minutes at room temperature with repeated mixing to obtain even CFSE labeling of all cells. The labeling was stopped by adding an equal volume of complete RPMI medium and removal of the solution from the cells by centrifugation. The cells were plated in round-bottomed 96-well cell culture plates at concentrations of 1 × 10^6^ cells per well and restimulated with Aβ42 peptide, anti-CD3, or ConA for 6 days. After being harvested, cells were resuspended in fluorescence-activated cell sorting buffer (PBS/1% bovine serum albumin/0.1% NaN_3_) and stained with an allophycocyanin-labeled mouse antihuman CD4 antibody or phycoerythrin-cyanine 7-conjugated mouse antihuman CD8 antibody (Tonbo Biosciences, San Diego, CA, USA). Fluorescence of the cells was measured using a BD Accuri C6 Plus flow cytometer and analyzed with CFlow Plus (BD Biosciences).

### Immunohistochemistry of mouse brain

Brain sections of triple-transgenic (3xTg)-AD mice were stained with plasma samples derived from the DNA of Aβ42-immunized rhesus monkeys and the control monoclonal antibody 6E10 (mouse antihuman Aβ42). For antigen retrieval, the sections were incubated in 70% formic acid prior to staining. Brain sections were incubated with the plasma samples (dilution 1:20) overnight at 4 °C. Antibody binding was detected with an HRP-conjugated rabbit antimonkey IgG antibody, which was detected with a Poly-HRP tertiary antibody followed by Alexa Fluor 488-tyramide signal amplification (Life Technologies, Carlsbad, CA, USA). Sections were counterstained with 4′,6-diamidino-2-phenylindole. Images were acquired using a Zeiss Axio Scan slide scanner and analyzed with the Zen lite software package (Carl Zeiss Microscopy, Jena, Germany).

### Statistics

For statistics (unpaired *t* test with two-tailed *p* values, Mann-Whitney *u* test, column statistics) we used Prism for Windows version 6 software (GraphPad Software, La Jolla, CA, USA). *p* Values ≤0.05 were considered significant.

## Results

### Blood work

Blood from the eight animals used in this study was analyzed before the first vaccination, following the fourth vaccination, and 12 weeks past the sixth vaccination for signs of inflammation (CBC) or signs of metabolic changes (blood chemistry). No changes were observed (data not shown).

### Evaluation of the injection sites

**Fig. 2:**
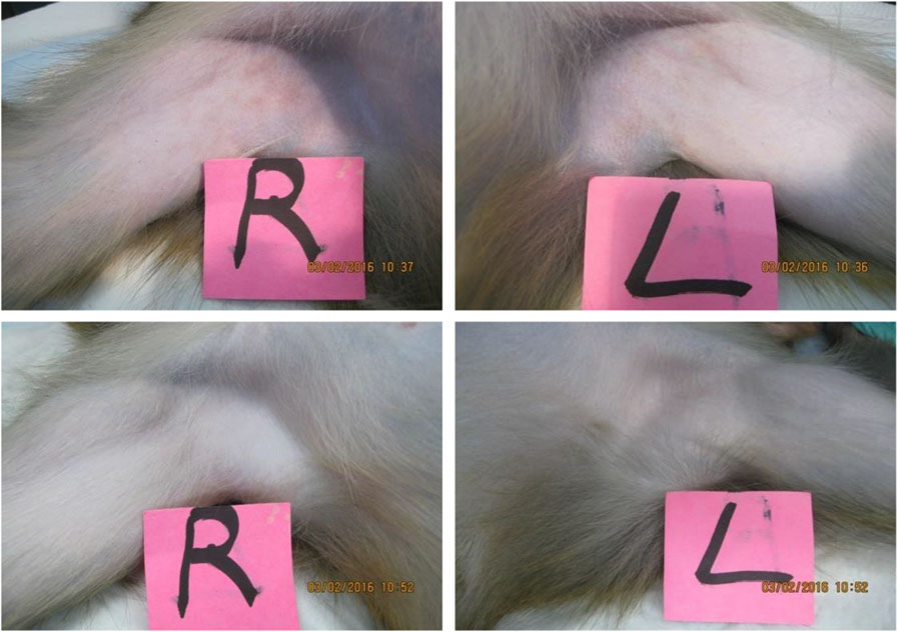
Photographs showing the axillary area of two animals that received DNA amyloid-β peptide 1–42 immunizations delivered with the gene gun 2 weeks past the second immunization. *L* Left side, *R* Right side

DNA Aβ42 injection via gene gun was delivered to the shaved skin area of the axillae. Adverse effects (redness, itching, and inflammation) were not observed in the immunized animals following the six immunization time points. The characteristic appearance of the skin injection sites is shown for two macaques in Fig. 2. Pictures were taken 14 days after the second immunization prior to the third immunization time point. Shown are the upper inner arms and arm axillae from two animals.

### Antibody immune response in DNA Aβ42-immunized rhesus macaques

#### Antibody levels and antibody titers

**Fig. 3:**
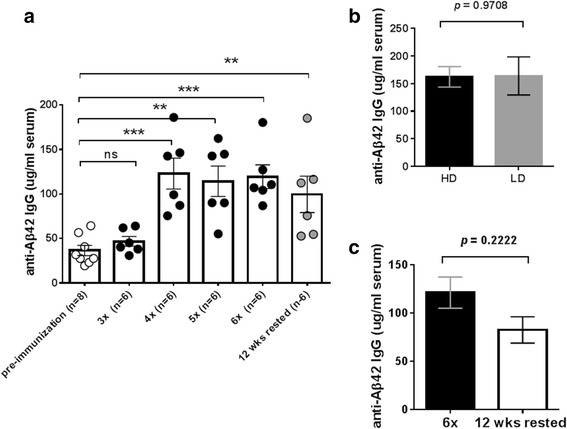
Antibody production in rhesus macaques after the different DNA amyloid-β peptide 1–42 (Aβ42) immunization time points. **a** Anti-Aβ42 antibody levels in DNA Aβ42-immunized rhesus monkeys (*n* = 6) are shown for blood drawn prior to the immunizations, from blood of animals immunized three, four, five, and six times, and from blood drawn following a 3-month rest period. **b** Antibody levels in animals that had received the high dose (16 μg of DNA per immunization time point, *black bar*) or the low dose (8 μg of DNA per immunization time point, *gray bar*) did not differ significantly following the sixth immunization time point. **c** Antibody levels declined slightly with the 3-month rest period

Two groups of rhesus macaques (*n* = 3/group) received two different doses of the DNA Aβ_1–42_ trimer vaccine consisting of 8 or 16 μg of DNA. These doses were extrapolated from our previous studies in mouse models and increased respectively based on the larger body size. We also used the 8- or 16-μg DNA doses in another set of experiments in which we analyzed the immune responses to DNA Aβ42 immunization in aged New Zealand White rabbits [34]. Antibody levels and total antibody amounts were measured for anti-Aβ42 antibodies of the IgG, IgA, and IgM isotypes. In blood samples taken prior to the first immunizations, baseline levels of anti-Aβ42 antibody levels were found (36.46 ± 16.24 μg/ml plasma, *n* = 8). Antibody levels increased to mean antibody levels of 46.78 ± 13.41 μg/ml plasma after three immunizations (*p* = 0.0383), to 114.2 ± 41.62 μg/ml plasma after five immunizations, and to 119.4 ± 32.55 μg/ml plasma after six immunizations (Fig. 3a). No significant differences were found between the low-dose group (8 μg of DNA per immunization) and the high-dose group (16 μg of DNA per immunization) (Fig. 3b). Antibody levels declined slightly to 99.53 ± 50.09 μg/ml (*n* = 6, *p* = 0.558) after a resting period of 3 months following the sixth immunization, so that the antibody half-life can be estimated as 4 months.

**Fig. 4:**
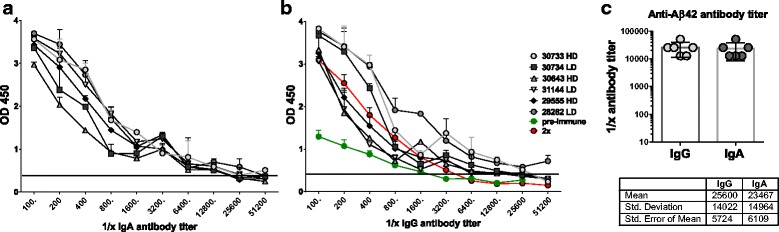
Antibody titers in the rhesus monkeys immunized six times. Serial dilutions of plasma samples from the six immunizations are shown. Immunization time points were tested for binding to amyloid-β peptide 1–42 (Aβ42) peptide in two-step enzyme-linked immunosorbent assays. **a** Antibody titers for anti-Aβ42 antibodies of the immunoglobulin A (IgA) isotype. **b** Antibody titers for antibodies of the IgG isotype. For comparison with antibody titers prior to the immunizations and to antibody titers following the second immunizations, these titers were included in the graph of the IgG titer analysis (*red* and *green lines*). Individual animals are shown with specific symbols as indicated on the figure. Animals receiving the high-dose (16 μg of DNA per immunization) and low-dose (8 μg of DNA/immunization) immunizations are indicated by HD and LD, respectively. **c** Comparison of IgG and IgA antibody titers in the immunized monkeys. Mean, SD, and SEM values are given in the table below the graph

Antibody titers for anti-Aβ42 IgG and IgA antibodies were determined from blood drawn after the sixth immunization time point. Plasma dilutions of 1:100 to 1:51,200 were tested for binding to Aβ42 peptide. The comparison with antibody levels prior to the immunizations is indicated by the green line in Fig. 4, and the comparison with antibody levels after two immunizations is indicated by the red line. Final antibody titers were calculated as the reciprocal of the highest serum dilution that gave a reading twice the baseline of an OD_450_ of 0.2. Both antibody isotypes showed titers greater than 1:20,000; a titer of 25,600 ± 14,022 was found for anti-Aβ42 IgG antibody, and a titer of 23,467 ± 14,964 was measured for anti-Aβ42 IgA antibody (Fig. 4).

#### Antibody isotypes and epitopes

**Fig. 5:**
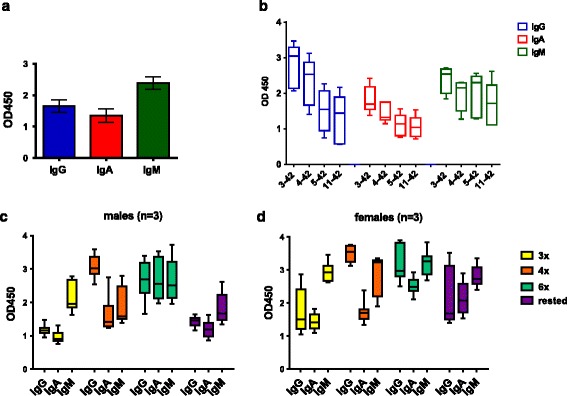
Antibody isotypes of anti-Aβ antibodies produced in the Rhesus monkey. **a** The humoral immune response generated anti-Aβ IgG, - IgM, and - IgA antibodies in the immunized monkeys. Plasma samples had been used in a 1:1000 dilution. **b** Binding of the different antibody isotypes to truncated Aβ_*x*–42_ peptides was tested from plasma after six immunizations (dilution 1:500). **c**, **d** Anti-Aβ42 IgG, -IgA, and -IgM antibody isotype responses were compared for different time points (*3x yellow bars*, *4x orange bars*, *6x light green bars*, and *three months rest purple bars*) in male C and female D Rhesus macaques. Results are shown in box & whiskers graphs from minimum to maximum values. Median levels were indicated with the *horizontal line*, the Standard Error Mean (SEM) is shown for all columns above and below the boxes

The humoral immune response consists of four antibody isotypes: IgM, IgG, IgE, and IgA. Among the four IgG subclasses in humans, rhesus monkeys possess three of them: IgG1, IgG2, and IgG4. We analyzed the binding of antibody isotypes IgM, IgG, and IgA to Aβ_1–40_ and Aβ_1–42_ peptides as well as to a panel of truncated Aβ42 peptides (Aβ_3–42_, Aβ_4–42_, Aβ_5–42_, and Aβ_11–42_). Anti-Aβ42 antibodies of all three isotypes were produced upon DNA Aβ42 vaccination in the rhesus monkeys (Fig. 5a).

High binding to Aβ_3–42_ and Aβ_4–42_ was observed for anti-Aβ42 IgG antibodies to Aβ_3–42_ and Aβ_4–42_, as well as good binding to Aβ_5–42_ and Aβ_11–42_. Antibodies of the IgA isotype bound similarly but less strongly to the truncated Aβ_*x*–42_ peptides. Good antibody binding to the truncated Aβ42 peptides was found for anti-Aβ antibodies of the IgM isotype (Fig. 5b). Next, we studied the development of the different isotype immune responses in the rhesus monkeys. Differences were found between the sexes of animals, with higher antibody responses seen in the females (Fig. 5c). Between the third and fourth immunizations, a strong boost of antibody production was found with highly increased levels of anti-Aβ42 antibodies of the IgG isotype. Anti-Aβ42 antibodies of the IgA isotype increased only slightly at the fourth immunization time point, but a large boost of an IgA antibody response was found with the sixth immunization. Antibody levels of all isotypes declined with the 3-month rest period, with a larger decline seen in the male monkeys.

**Fig. 6:**
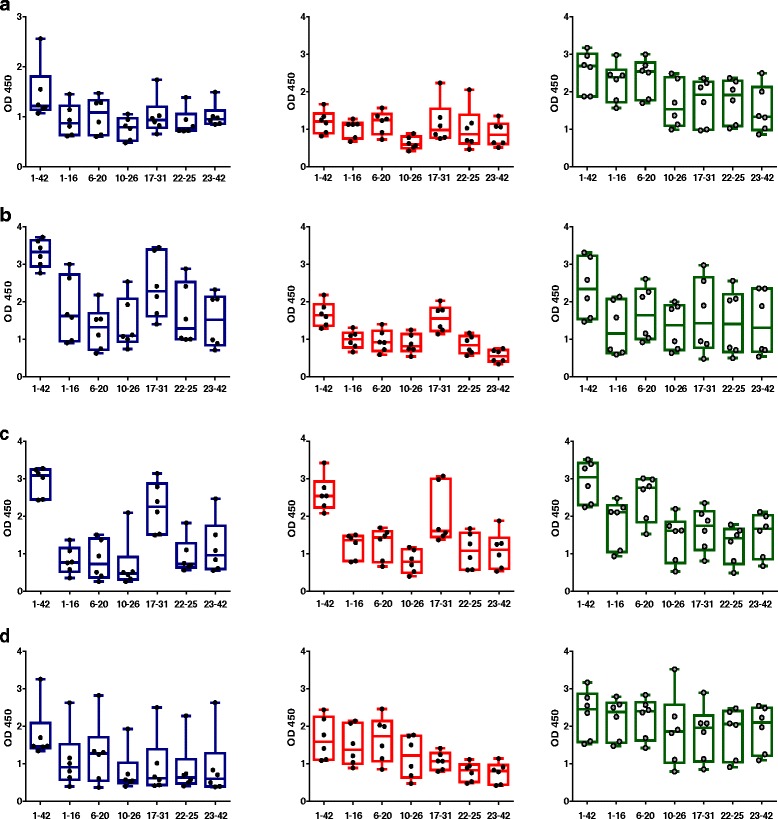
Amyloid-β peptide 1–42 (Aβ42) epitope binding of immunoglobulin G (IgG), IgA, and IgM isotypes at different time points of the immunization schedule. A panel of shorter Aβ peptides (Aβ_1–16_, Aβ_6–20_, Aβ_10–26_, Aβ_17–31_, Aβ_22–35_, and Aβ_23–42_) was used to detect linear epitope binding of antibodies generated in the animals after DNA Aβ42 immunization. **a** Aβ epitope binding pattern of IgG, IgA, and IgM antibodies observed after three immunizations. **b** Epitope binding pattern after four immunizations. **c** Epitope binding pattern found with six immunization time points. **d** Aβ epitope binding of antibodies of the IgG, IgA, and IgM isotypes after the 3-month rest period. Results are presented in box-and-whisker graphs with minimum to maximum values. Median levels are indicated by the *horizontal lines*, and the SEM is shown for all columns above and below the boxes. Each *circle* within the bars represents the value for one individual monkey that had received active DNA Aβ42 immunizations. IgG antibodies are indicated in *blue*, IgA antibodies are indicated in *red*, and IgM antibodies are indicated in *green. OD*_*450*_ 450-nm Optical density

For characterization of the linear anti-Aβ-specific B-cell epitopes, we used a panel of shorter Aβ peptides spanning the Aβ_1–42_ sequence: Aβ_1–16_, Aβ_6–20_, Aβ_10–26_, Aβ_17–31_, Aβ_22–35_, and Aβ_23–42_. In a direct ELISA, antibody binding of the IgG, IgA, and IgM isotypes to the shorter Aβ peptides was determined from blood samples following the third, fourth, and sixth immunizations and in the blood samples drawn 12 weeks past the sixth immunization (rested state) (Fig. 6). Consistent with the strong boost of antibody production following the fourth immunization, antibody binding to full-length Aβ_1–42_ as well as the different linear Aβ peptides increased, showing strong reactivity to all the shorter epitopes and highest binding of IgG antibodies to the midregion epitope Aβ_17–31_ (Fig. 6b). With the sixth immunization, a clear boost of IgA antibodies was detected again, with the highest binding to full-length Aβ_1–42_ and the midregion peptide Aβ_17–31_ (Fig. 6c). IgM antibodies in blood samples from monkeys immunized six times with DNA Aβ42 bound strongly to full-length Aβ_1–42_ and Aβ_6–20_. With the 3-months rest period following the sixth immunization, antibody binding of all isotypes (IgG, IgA, and IgM) and to all Aβ epitopes declined (Fig. 6d).

**Fig. 7:**
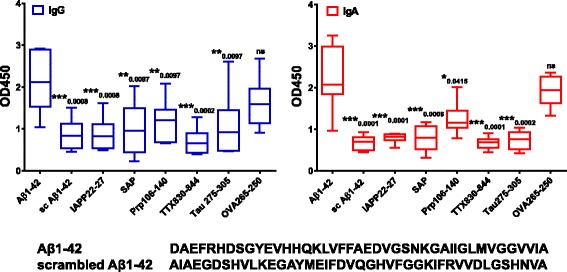
Specificity of antibodies in plasma of DNA amyloid-β peptide 1–42 (Aβ42) immunized rabbits to Aβ_1–42_. Plasma samples from the sixth immunization time point of the six immunized rhesus monkeys were tested for binding to a variety of different peptides (Aβ_1–42_, scrambled Aβ_1–42_, islet amyloid protein [IAPP], serum amyloid P component [SAP], prion protein fragments [Prp], tau peptide repeat 2 domain, tetanus toxin peptide [TTX], ovalbumin peptides [OVA]). Plasma was diluted 1:500 for these assays. In the comparison of binding to Aβ_1–42_, significant differences were found for binding of immunoglobulin G (IgG; *blue bars*) and IgA (*red bars*) antibodies in plasma to the distinct peptides tested (indicated by *, **, *** *p* values above the bars). Differences in IgG and IgA antibody binding in plasma samples from the immunized monkeys to nonrelated OVA peptides compared with binding to Aβ_1–42_ were nonsignificant (Mann-Whitney *p* values of 0.1246 and 0.5532, respectively). *OD*_*450*_ 450-nm Optical density

Antibody specificity was tested by comparing the binding of antibodies in plasma from the immunized rhesus monkeys to Aβ_1–42_ peptide, scrambled Aβ_1–42_ peptide, islet amyloid protein peptide (IAPP_22–27_), SAP, Prp_106–140_, tau peptide repeat 2 domain (275–305), TTX_830–844_, OVA_257–264_, OVA_265–280_, and OVA_323–339_. Very low binding was found for the scrambled Aβ_1–42_ peptide, which is the optimal negative control owing to the presence of all amino acids present in Aβ_1–42_ in a randomly altered sequence. It showed that the specific amino acid sequence is critical for antibody binding (*p* values of 0.0008 for IgG plasma antibodies and 0.0001 for IgA plasma antibodies in comparison to binding to Aβ_1–42_ by Mann-Whitney *t* test) (Fig. 7). Amino acid sequences for the two peptides, Aβ_1–42_ and scrambled Aβ_1–42_ are illustrated in Fig. 7. Similar low binding was found for IgG and IgA antibodies in plasma from the immunized animals to IAPP, SAP, Prp, and TTX peptides (*p* values ranging from 0.0002 to 0.0415 in the comparison of binding to Aβ_1–42_) (Fig. 7). High binding was found for plasma antibodies of IgG and IgA isotypes to all OVA peptides tested (data not shown; only OVA_265–280_ is shown in Fig. 7). A literature search showed that blood of 90% of adult human donors contained IgG antibodies against OVA as a food-derived antigen [35], which might explain the high level of cross-reactivity observed.

### Analyses of T-cell responses in the immunized rhesus macaques: cytokines and proliferation

**Fig. 8:**
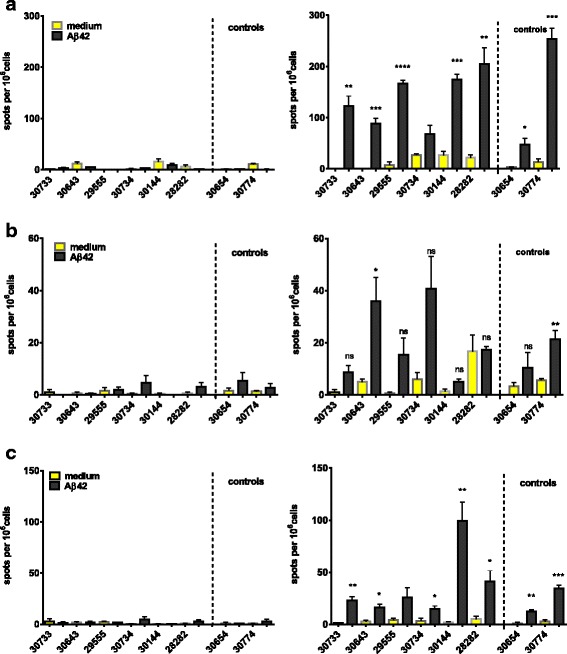
Interferon (IFN)-γ, interleukin (IL)-17, and IL-4 Enzyme-Linked ImmunoSpot assays for amyloid-β peptide 1–42 (Aβ42) peptide restimulated peripheral blood mononuclear cell cultures of DNA Aβ42-immunized rhesus monkeys. The first column shows the number of IFN-γ-, IL-17-, and IL-4-secreting cells after three immunization time points (48 h in cell culture, medium controls, and Aβ42 peptide restimulation). The second column provides results from the same analyses after the sixth immunization time point. Individual rhesus monkeys are indicated with numbers on the *x*-axis of all graphs. The *y*-axis of all graphs shows the number of cytokine-secreting cells (spots) per 10^6^ cells. Increased numbers for all three cytokines were found in the immunized animals as well as in the nontreated control animals. Therefore, the cytokine secretion was considered as nonspecific and not due to the DNA Aβ42 immunization. **a** IL-17-secreting cells. **b** IFN-γ-secreting cells. **c** The number of IL-4-secreting cells per 10^6^ splenocytes. * *p* < 0.05, ** *p* < 0.005, *** *p*  < 0.001, and **** *p* < 0.0001

**Fig. 9:**
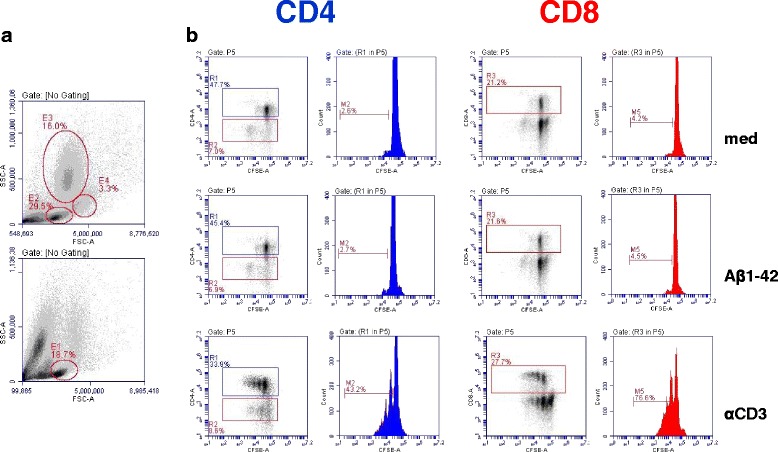
CD4 and CD8 T-cell proliferation in amyloid-β peptide 1–42 (Aβ42) peptide restimulated peripheral blood mononuclear cell cultures. **a** Mononuclear blood cells were separated on the basis of forward scatter (FSC) and side scatter (SSC). The upper histogram shows the pattern in fresh blood, and the lower histogram shows the cell populations after 6 days in culture. Gates for the carboxyfluorescein succinimidyl ester (CFSE) assay were set on lymphocytes (E1). **b** T-cell proliferation was analyzed using a CFSE dilution assay. Events were gated on CD4- and CD8-positive cells, and cytometric histograms are shown for medium controls, Aβ42 peptide restimulated cultures, and proliferation after anti-CD3 antibody stimulation. All samples were run in triplicates. No T-cell proliferation in response to Aβ42 peptide restimulation was found

IFN-γ, IL-17, and IL-4 secretion was determined by an ELISPOT assay for all of the animals (*n* = 8) 1 month following the third immunization and 3 weeks following the sixth immunization from peripheral blood mononuclear cells (PBMCs). After the third immunization, no IFN-γ-, IL-17-, or IL-4-secreting cells were detected after Aβ42 peptide restimulation, whereas high numbers of spots were found in wells that had been stimulated with ConA or anti-monkey CD3 (Fig. 8a and data not shown). Further T-cell responses were determined in the six immunized and two control animals 3 weeks following the sixth DNA Aβ_1–42_ immunization by ELISPOT assays for IFN-γ, IL-17, and IL-4 (Fig. 8b) and a CFSE proliferation assay with PBMCs (Fig. 9). Increased numbers of IFN-γ-, IL-17-, and IL-4-secreting cells were found in all the animals, including the control animals, which had not been immunized. The number of IFN-γ-secreting cells was highly significant in all monkeys except one, including the control animals (*p* < 0.05, *p* < 0.005, *p* < 0.0001). One of the control animals actually had the highest number of IFN-γ-secreting cells (253.3 ± 37.23 spots/10^6^ cells). Similarly, the numbers of IL-17 secreting cells was elevated. A significant increase was found for one of the immunized rhesus macaques (*p* = 0.0269) and for one of the control animals (*p* = 0.001). The number of IL-4-secreting cells was also significantly increased in all the animals except one (*p* < 0.05 and *p* < 0.005) (Fig. 8b). No proliferation of CD4 or CD8 T cells was found in the wells that had been restimulated with Aβ42 peptide for 6 days (Fig. 9). Good proliferation was found for the PBMCs in the control wells of each rhesus macaque that had been restimulated with ConA or an anti-CD3 antibody, confirming the viability of the cells.

**Fig. 10:**
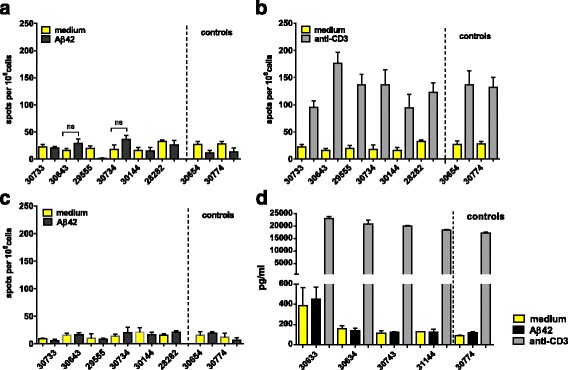
Interleukin (IL)-17 and IL-4 Enzyme-Linked ImmunoSpot (ELISPOT) assays, as well as interferon (IFN)-γ enzyme-linked immunosorbent assay (ELISA) from amyloid-β peptide 1–42 (Aβ42) peptide-restimulated peripheral blood mononuclear cell cultures of DNA Aβ42-immunized rhesus monkeys after a 3-month rest period. IL-17- and IL-4-secreting cells were analyzed in an ELISPOT assay (**a–c**). IFN-γ secretion was tested with a cytokine ELISA (**d**). The individual rhesus monkeys are indicated with numbers on the *x*-axis of all graphs. **a–c** The *y*-axis shows the number of cytokine-secreting cells (spots) per 10^6^ cells. **d** The *y*-axis shows the amount of IFN-γ found (expressed in picograms per milliliter of culture supernatant)

Cytokine secretion after Aβ42 peptide restimulation was again examined 3 months following the sixth immunization (Fig. 10a–d). No significant increase was found for the numbers of IL-17- and IL-4-secreting cells (Fig. 10a, b). Upon anti-CD3 stimulation, PBMCs from all monkeys showed increased spots of IL-17-secreting cells, demonstrating the viability of the cells in culture (Fig. 10c). IFN-γ was analyzed in an ELISA using cell culture supernatants of PBMCs that had been cultured for 48 h and 96 h with medium alone or restimulated with Aβ_1–42_ peptide or ConA or anti-CD3 antibody. No Aβ42-induced IFN-γ secretion was found (Fig. 10d). One animal (30633) had higher levels of IFN-γ in the cell culture supernatant in the medium control wells as well as in the Aβ42 peptide-restimulated well. Owing to the higher levels already in the control supernatant and no significant increase in the Aβ42 peptide-restimulated cultures, the IFN-γ production is nonspecific and not due to the DNA Aβ42 immunizations.

### Antibodies generated in rhesus macaques bind to amyloid plaques in an AD mouse model

**Fig. 11:**
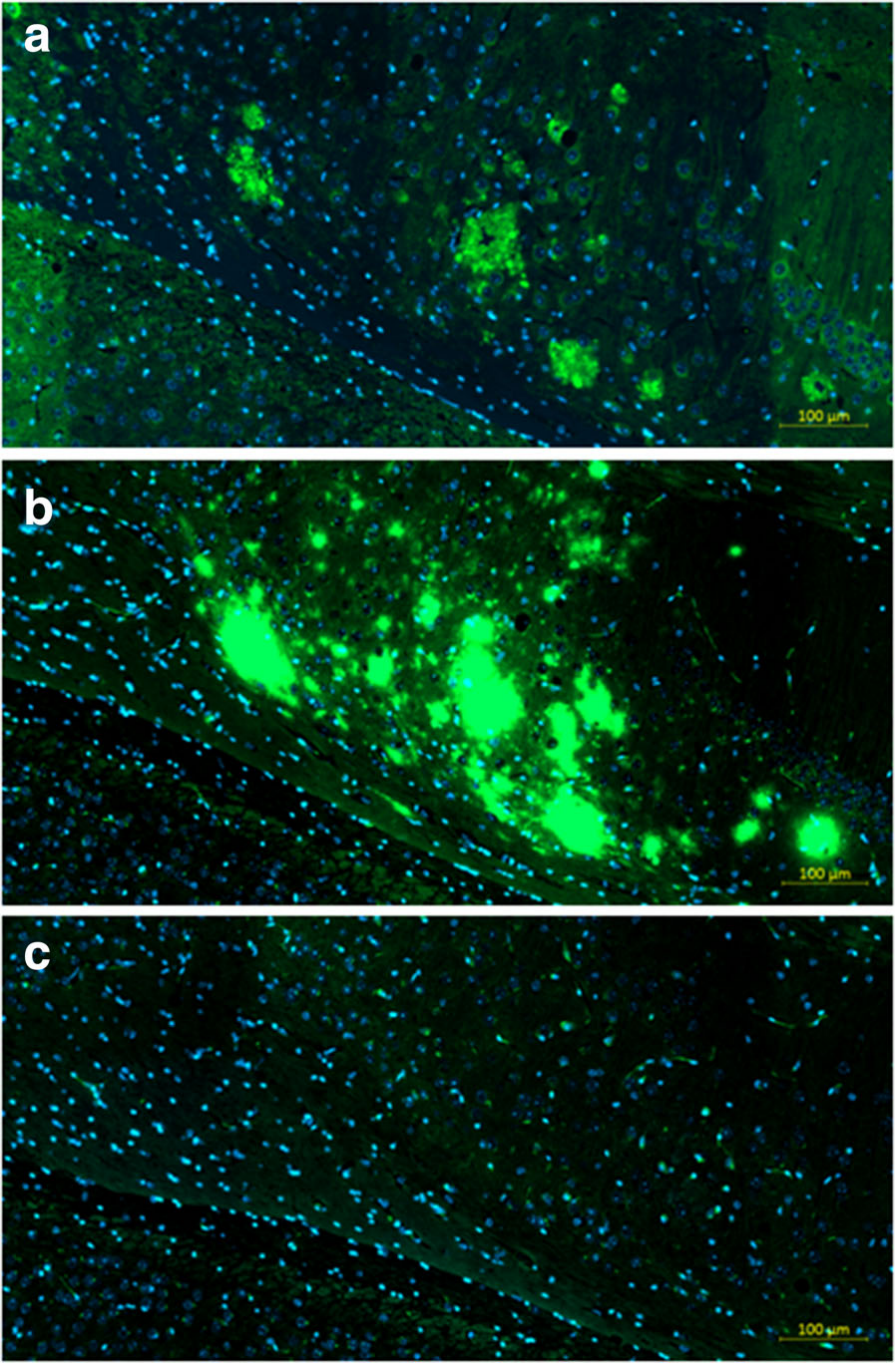
Antibodies from DNA amyloid-β peptide 1–42 (Aβ42)-immunized monkeys stain amyloid plaques containing human Aβ42 peptides in brain sections of triple-transgenic Alzheimer’s disease (3xTg-AD) mice. **a** Plaque staining in hippocampal area of 3xTg-AD mouse with plasma from animal 28282. **b** Control staining in parallel section with commercial anti-Aβ42 antibody (6E10). **c** No staining in parallel section that was incubated with the secondary antibody and detection reagents only. Scale bar indicates 100 μm

The specificity of the antibody response in the DNA Aβ42-immunized rhesus monkeys was further tested for staining of senile plaques containing human Aβ42 in an AD mouse model. The staining of brain sections from a 3xTg-AD mouse [B6;129-*Psen1* ^*tm1Mpm*^ Tg(APPSwe,tauP301L)1Lfa/Mmjax; The Jackson Laboratory, Bar Harbor, ME, USA] with plasma from the monkeys that had received DNA Aβ42 immunizations showed clear staining in the typical area in which amyloid plaques are present in this AD mouse model: the subiculum of the hippocampus (Fig. 11a). In the comparison with a commercial anti-Aβ42 antibody (6E10, shown in Fig. 11b), the stained areas are overlapping. No staining was found in a brain section that was stained only with the secondary antibody and detection reagents (Fig. 11c). Thus, antibodies generated in the immunized rhesus monkeys upon DNA Aβ42 vaccination detected human Aβ42 in brains of the AD mouse model.

## Discussion

DNA Aβ42 trimer immunization with a two-plasmid system delivered via gene gun into the skin led to good antibody responses in the rhesus monkey. The results described in this report were very similar to the findings we published for experiments with mice (wild-type and AD mouse models) and rabbits [18–25, 34]. The anti-Aβ42 IgG antibody levels produced in the six monkeys reached mean values of 120 μg/ml plasma after six immunizations, as well as mean antibody titers of 1:25,000. The antibody half-life was estimated as 4 months. The data show that intradermal DNA Aβ42 immunization is a successful route to trigger an antibody response against a self-antigen in large mammals. In regard to the antibody titers found in this study, it is important to note that antibody titers of 1:2000 found in patients in the AN1792 clinical trial had been shown to be sufficient for removal of Aβ from the brain and significant reduction of the plaque load [8]. Thus, the antibody levels found here following DNA Aβ42 immunization in rhesus macaques (1:25,000) are highly likely to be effective for removal of amyloid from the brain in patients with AD.

Anti-Aβ antibodies produced after DNA Aβ42 immunization detect a wide variety of epitopes on the Aβ_1–42_ peptide, including N-terminal truncated Aβ isoforms, and should thus be efficient also in removing N-truncated Aβ_*x*–42_ species for which it had been shown in a case study that they remained in brains from patients who had received the AN1792 Aβ42 peptide vaccine producing antibodies mainly against the B-cell epitope in Aβ_1–42_, which is Aβ_1–15_ [36, 37].

In work done at a different laboratory where researchers developed an epitope vaccine containing three copies of Aβ_1–11_ with inclusion of eight nonself T-helper epitopes into the DNA construct (3 × -Aβ_1–11-Thep_), two groups of monkeys received three intramuscular immunizations with 0.4 mg and 4 mg of the DNA vaccine, and antibody titers of 1:2500 (0.4 mg) and 1:6000 (4 mg) were found [26]. Intramuscular DNA immunization compared with intradermal DNA immunization uses much higher doses of DNA and is considered more prone to cellular and potentially inflammatory immune responses. Cellular anti-Aβ42 immune responses were not found in this vaccine, because it contains only the B-cell epitope, but high doses of DNA were used to mount an antibody response. This group optimized the vaccine, and in a second group of immunized macaques receiving the optimized vaccine, mean antibody titers reached 1:6050 and 1:9680 (week 50), respectively, which declined quickly in the following 10 weeks (week 60, 1:1375 and 1:3190, respectively [27]). Monkeys immunized with the epitope vaccine study produced only IgG antibodies, which is likely due to the lack of T-cell help during the generation of an antigen-specific immune response.

In a study done at another laboratory, the analyses of groups of aged (10–26 years old) and juvenile (1–2 years old) macaques, which had received a total of five Aβ42 peptide (100 μg/time point) immunizations with monophosphoryl lipid A as an adjuvant, it was found that only the juvenile animals developed a strong and sustained antibody response [38]. Despite the low levels of antibodies in the aged animals, the analysis of cerebrospinal fluid Aβ42 levels showed a positive correlation between Aβ42 and anti-Aβ42 antibody levels, which was significant for the subgroup of aged animals but not for the subgroup of juvenile animals. The findings imply that even with a low antibody response upon Aβ42 immunization, the effects on Aβ42 removal from the brain are measurable. The adult cohort (6–10 years old) analyzed in this study developed a strong antibody response in all animals, and we predict, on the basis of data derived from the use of this immunization protocol in aged mice and rabbits, that DNA Aβ42 immunization will also lead to strong humoral immune responses in patients with AD [24, 34].

The strong IgA antibody response found in our study and in our previous analysis to the immune responses in New Zealand White rabbits [34] was unexpected. In a different DNA immunization approach in which rhesus monkeys received a protective DNA vaccine against simian immunodeficiency virus (SIV) infection, which was delivered on DNA-coated gold particles bombarded into the epidermis near the inguinal lymph node by using the PowderJect XR1 gene delivery device (PowderJect Vaccines/Pfizer, New York, NY, USA), a humoral immune response consisting of high levels of IgG and IgA antibodies was found [39, 40]. DNA doses were 20 μg of DNA/immunization/animal, and the mean SIV gp120-specific antibody titer for all monkeys following the fourth DNA dose was 1:9142. Even though this is a completely different approach and intended to induce a mucosal immune response, the comparison with this study is of interest for two reasons: one is the finding of high levels of IgA antibodies, and the second is the comparison of antibody titers. Considering that the immunization with a plasmid DNA encoding a virus protein (SIV/17E-Fr gag-pol-env), which is a foreign and pathogenic antigen, resulted in an antibody titer of 1:10,000, which was protective in the macaques against SIV infection, and an antibody titer of 1:25,000 against a self-antigen (Aβ_1–42_), presented in our study, is indicative of a good immune response.

During the humoral antibody responses, antibody class switch recombination (CSR) to produce antibodies with the IgA isotype is controlled by cytokine signals. The major cytokine signal for α-CSR is transforming growth factor (TGF)-β together with additional contributions from IL-2, IL-4, IL-5, IL-6, and IL-10 [41]. Importantly, the cytokines IL-4, IL-5, and IL-10 are all indicative of a Th2 immune response, and IL-10 and TGF-β are produced by regulatory immune cells, which is consistent with our previous findings demonstrating a Th2/regulatory T-cell immune response following DNA Aβ42 immunization in the mouse model [20–24].

No cytokine secretion was found after three immunizations in rhesus monkeys that had received the MultiTEP-Aβ epitope vaccine (Vaxine Pty, Adelaide, Australia) [26], which is consistent with the findings in our study using a full-length DNA vaccine. Elevated cytokine levels were found at the sixth immunization time point in our study, but they were considered nonspecific because the control animals had increased numbers of cytokine-producing cells as well. In the parallel analyses of T-cell proliferation, no proliferation of CD4 or CD8 T cells was found for cells that had been restimulated with Aβ42 peptide. This lack of T-cell proliferation in response to peptide restimulation is a second argument that the low though significantly increased numbers of cytokine-secreting cells were not in response to the Aβ42 peptide restimulation, and thus nonspecific. The numbers of IL-17-secreting cells went down again after a 3-month rest period, and no IFN-γ was found in Aβ42 peptide-restimulated PBMC cultures at this time point. Altogether, we conclude that DNA Aβ42 immunization does not lead to inflammatory T-cell responses in NHPs, similar to our previously described results in mice and in rabbits [20–24, 34].

## Conclusions

DNA Aβ42 immunization led to a good humoral immune response in rhesus macaques that had received a total of six immunizations. High titers of anti-Aβ42 IgG and IgA antibodies were found, which might complement each other in their effect to remove excess amyloid from the brain. Anti-Aβ antibodies produced after DNA Aβ42 immunization detect a wide variety of epitopes on the Aβ_1–42_ peptide, including N-terminal truncated Aβ isoforms. Antibodies produced after DNA Aβ42 immunization in rhesus monkeys are Aβ42-specific because low binding to a panel of distinct peptides tested was found. Anti-Aβ antibodies produced after DNA Aβ42 immunization detect plaques consisting of Aβ peptides of human type in the brain of an AD mouse model. No indications for an inflammatory cellular immune response (IFN-γ and IL-17) were observed in mice [20–24], rabbits [34], or NHPs as shown in our study. On the basis of these data, we predict a positive outcome, with good humoral immune responses and no inflammation, in patients with early AD in a clinical trial using full-length DNA Aβ42 trimer vaccination.

## Abbreviations

3xTg: Triple-transgenic
AD: Alzheimer’s disease
Aβ42: Amyloid-β peptide 1–42
CBC: Complete blood count
CFSE: Carboxyfluorescein succinimidyl ester
ConA: Concanavalin A
CSR: Class switch recombination
ELISA: Enzyme-linked immunosorbent assay
ELISPOT: Enzyme-Linked ImmunoSpot
FSC: Forward scatter
HD: High dose
IAPP: Islet amyloid protein peptide
IFN: Interferon
Ig: Immunoglobulin
IL: Interleukin
LD: Low dose
NHP: Nonhuman primate
OD_450_: 450-nm Optical density
OVA: Ovalbumin
PBMC: Peripheral blood mononuclear cell
Prp: Prion protein
SAP: Serum amyloid component P
SIV: Simian immunodeficiency virus
SSC: Side scatter
TGF: Transforming growth factor
TTX: Tetanus toxin
